# Enacting Identity and Transition: Public Events and Rituals in the University (Mexico and South Africa)

**DOI:** 10.1007/s11024-015-9287-0

**Published:** 2015-12-28

**Authors:** Wil G. Pansters, Henk J. van Rinsum

**Affiliations:** Faculty of Social and Behavioural Sciences, Department of Cultural Anthropology, Utrecht University, Utrecht, The Netherlands; Faculty of Arts, University of Groningen, Groningen, The Netherlands

**Keywords:** Public events, Ritual, Transition, Identity, University

## Abstract

On the basis of ethnographic and historical material this article makes a comparative analysis of the relationship between public events, ceremonies and academic rituals, institutional identity, and processes of transition and power at two universities, one in Mexico and the other in South Africa. The public events examined here play a major role in imagining and bringing about political shifts within universities as well as between universities and external actors. It shows how decisive local histories and constituencies are in mediating and transfiguring identity projects initiated from above.

## Introduction

On May 13, 1994, a baroque room inside the building complex of the Benemérita Universidad Autónoma de Puebla (BUAP) was the scene of a ceremonial event. Rector José Doger had invited university authorities and regional dignitaries, such as the state governor, the mayor and representatives of business organizations, to be present at the official launch of the university’s first long-term strategic development plan. Following the rector’s speech, Governor Bartlett of the state of Puebla lauded the university’s efforts to embark on a course that would transform the institution.

On February 20, 2004, the formerly Afrikaner University of Stellenbosch (SU) awarded an honorary degree to South African President Thabo Mbeki. In his acceptance speech, Mbeki said the following in Afrikaans, the disputed language at SU, “When I leave this Oak-city [i.e. Stellenbosch], I will travel our country and I will reach for the highest mountains. And there I will proudly say to the world, that I too have become, honoris causa, a scholar of Matieland.”^1^

On May 27, 2015, a ceremony took place on SU’s campus to remove the commemorative plaque of former Prime Minister Hendrik Verwoerd, architect of South African apartheid and professor at the University of Stellenbosch, from the Accounting and Statistics Building, which was formerly also known as the HF Verwoerd Building. The vice-chancellor of the university as well as Verwoerd’s grandson—who had rejected apartheid many years ago and become a member of the ANC—were present. This symbolically and emotionally charged public event ended with the singing of the national anthem.^2^

These public events can be seen as instances of the enactment of new institutional narratives that result from, and are constitutive of transitional processes in two different but comparable countries and circumstances. In terms of political economy both Mexico and South Africa had initiated neoliberal reform during the 1990s and were particularly focused on achieving a prominent position in the global competitive picking order. As for political change, both countries had left behind authoritarian systems—one based on authoritarian presidentialism, the other on racial exclusion—and were moving towards transition and democratization (For Mexico, see Harvey 1993; Harvey & Serrano 1994; Middlebrook 2004; Araujo 1996. For South Africa, see Bond 2000; Lodge 2002).

In this article we examine a series of public events staged as university ceremonies and rituals and attempt to understand their significance for political and symbolic transitions and identity formation in Mexican and South African higher education, as well as for processes of change in the society at large. We explore their political meaning and effectiveness. We ask whether and how organizations carve out distinct identities through ritual and ceremonies as standardized and repetitive symbolic behavior (Kertzer 1988: 9). We argue that public events at the BUAP and SU, such as the granting of honorary doctorates, enacted and effectuated the re-imagination of organizational power and identity, and the symbolical articulation of the shifting political relations within the universities as well as between the universities and external actors within the society at large.

In what follows, we formulate a conceptual framework which helps us to analyze and frame our case studies. We then briefly sketch major developments in higher education in Mexico and South Africa as part of the broader neoliberal reform processes and the dismantling of authoritarian political structures. We present some crucial information about the BUAP and SU so as to better understand the context and history against which public events and ceremonies—including academic rituals—were played out. Rewriting the histories of these two universities through these public displays constitutes a key element of our analysis. Thereafter, we turn to the politics of institutional identity. We examine the construction of institutional narratives of identity that reposition the two universities vis-a-vis their own history and other social actors. We then look at how public events and ritualized practices play a constitutive role in redefining institutional identity. In the final section, we explore whether and how specific public events and practices at the BUAP and SU can be understood as constituting a comprehensive ritual of transition.

## A Conceptual Framework: Public Events, Academic Rituals and Transition

Anthropologists are traditionally concerned with the cultural dimensions of (political) transitions, since they are particularly interested in understanding the symbolic and discursive recasting of the political. Successful transitions appear to be intimately connected with the invention of historical or ethical discourses, the construction of particular symbols, and the enactment of diverse public events to forge new institutional identities. Equally, transitions involve the re-signification of existing symbols, ceremonies and rituals to make the new situation intelligible and legitimate.^3^

Initially, we set out to study these events and their relationship to broader processes of transition and identity (re)formation with the conceptual framework of ritual theory. After all, in the seminal works of Van Gennep and Turner, ritual is strongly associated with transition and change, sometimes even implying deep divisions and conflict. Van Gennep’s theory of rites of passage (1909) identified the common structure in rites that symbolize the transition from one position to another in families, groups, or society at large: separation, marginality, and aggregation. This emphasis on transition and transformation subsequently influenced the work of the English anthropologist Turner (1967), thereby creating something of a Van Gennep-Turner tradition, which made it possible to speak of “rituals of revolution” that embody historical rupture, the reconstitution of authority, shifting alliances, and the elaboration of new historical narratives. Since then, this concept has been applied to highly different social and cultural contexts. In his study of public rituals of the late French president François Mitterand, Abélès (1988) concludes that there is no difference in kind between modern political rituals and those of traditional societies that are pervaded by magico-religious meanings evoking such transcendental symbols as Nation, Republic and History. Rituals are thoroughly codified and solemn. According to Abélès, one of the key characteristics of a ritual is “dramatization, the acting out of performances that mobilize public support”; that is to say, in a combination of “spoken words, significant acts and manipulated objects” rituals dramatically enact (political) messages, processes and change (Abélès 1988: 393). We argue that university rituals and ceremonies studied here could best be seen as ‘representational rituals’ that symbolize significant features of social reality. More specifically, we consider the granting of honorary degrees as a specific academic ritual since it symbolically articulates the transition from one particular state to another (membership of an academic community). In other words, theorizing about ritual is helpful for our main goals.

However, many other public events and academic ceremonies we regard relevant for understanding the symbolic dimensions of institutional transitions and identities, such as the launch of the strategic development plan in Puebla and the removal of Verwoerd’s commemorative plaque in Stellenbosch, do not match the definitional logic of separation, marginalization/liminality, and (re-)aggregation, and hence can not properly be understood as rituals per se. They do not even seem to match Kertzer’s (1988: 9, 11) much broader definition of ritual as “socially standardized and repetitive” behavior “wrapped in a web of symbolism,” which grants rituals a dramatic quality that does not only define roles but also “provokes an emotional response.” While these events in Puebla and Stellenbosch were certainly dramatic, they were also unique rather than standardized and repetitive. Moreover, as an anonymous reviewer noted, we encountered definitional problems with the concept of ritual. With subtlety and irony Handelman (1998: 10), for example, observed that when it comes to abstract statements of definition, a “truly massive accumulation of studies on…‘ritual’ has remained ‘strangely uninformative – not only for purposes of comparison’, but also merely for getting an idea of what is being talked about.” A long list of ‘ad hoc and piecemeal’ nearly synonyms or variations of ‘ritual’ has emerged in much anthropological and historical writing, which makes a precise and clear use of the concept of ritual very difficult (Handelman 1998: 14). He therefore prefers to speak of “public events,” which are “locations of communication that convey participants into versions of social order in relatively coherent ways” (Handelman 1998: 15). Public events possess an intentionality and a capacity to be consequential for social life. In Handelman’s (1998: 16) eloquent wording, public events “…exist in the lived-in worlds of the participants…are graspable as such by external observers. Their mandate is to engage in the ordering of ideas, people and things…they are devices of praxis that merge horizons of the ideal and the real, to bring into close conjunction ideology and practice, attitude and action.” We believe that this more generic concept of public events better serves our purpose here, as it can accommodate the examination of events we deem important for the construction of new institutional narratives and identities. The latter are particularly pertinent in periods of wider societal transitions, when there is a need to ‘merge the horizons’ of the ideals of what the transition aspires to accomplish and the realities handed over by the (immediate) past. Our interest lies first and foremost in understanding how universities develop and adopt new strategic narratives and in how they publically display them.^4^ We believe that public events and specific rituals are ‘privileged points of penetration into other social and cultural universes,’ in our case meaning wider social and institutional transitions (Handelman 1998: 9).

While it is useful to distinguish between ritual and public event when discussing specific concrete events, we also find it challenging to investigate the critical distinction between individual rites of passage and broad political transitions (as in South Africa and Mexico). We are inspired by Desfor Edles’s (1998) cultural understanding of the entire post-Franco democratic transition in Spain as a comprehensive ritual, which in its turn encompasses more specific public events and rituals. Edles examines the Spanish democratic transition in terms of the phases of separation, liminality and reaggregation. Separation then refers to leaving behind an old social state, a detachment from an earlier point in time and social structure. During the liminal phase, the characteristics of the ritual subject are ambiguous. It may be peculiar to conceive a liminal phase of a transitional ritual, since “a political transition is by definition a ‘liminal’ stage between two distinct social states, and the goal of (political) transition is the successful putting behind of the old, and the consummation of the new social state” (Edles 1998: 24). Even so, she substantiates the symbolic representations of liminality by stressing the homogeneity and communality experienced by different political actors in post-Franco Spain. Key discursive symbols of this phase were ‘national reconciliation’ and ‘*convivencia*’ (literally, ‘living together’), which were articulated during critical consensual events. Drafting the Moncloa Pacts in October 1977 was such an event, where former enemies reconciled their differences and sought solutions to economic and political problems to accomplish the transition from *franquista* authoritarianism toward a democratic polity. In the Spanish case, the moment of reaggregation was epitomized by the drafting of the Constitution in 1978. Reaggregation marks the end of the ritual process of transition and involves a process of discursive closure. In this article we will examine whether this framework is helpful to us in understanding and conceptually ordering developments in the field of higher education, specifically in the cases of the BUAP and SU.

In sum, we hope to shed light on the imagination, representation and realization of political shifts *within* universities, their institutional narratives as well as the political relationships *between* universities and other societal actors by looking at public events, ceremonies, and particular academic rituals. In addition, we want to employ a framework that sees transition as a comprehensive ritual process that envelops the previous more specific events. In other words, in our analysis of transition and identity formation at the universities of Puebla and Stellenbosch, we apply a perspective that takes into consideration public events, ceremonies and rituals within universities as well as broad (democratic) transitions understood as comprehensive ritual, which in its turn encompasses the former. It is our hope that such an approach will provide insight into the complexities, ambiguities, and contradictions of the politics of institutional identity formation and transition that go beyond one-dimensional understandings.

## Historical Development of Institutional Identities at BUAP and SU

Since the beginning of the 1980s, a wave of neoliberal policies has affected the functioning of economies, political systems, and institutions in civil societies across the globe. In Mexico, the restructuring of state–society relations in the broadest sense of the term took off after the 1982 economic crisis. A new developmental project deepened and accelerated during the presidencies of Carlos Salinas (1988–1994), Ernesto Zedillo (1994–2000), and Vicente Fox (2000–2006). Government institutions and organizations highly dependent on public resources were drawn into a discourse on modernization, accountability, competition, quality management, efficiency and openness. The new development strategy encompassed economic restructuring by way of trade liberalization, the privatization of state-owned companies, hesitant political reform, new social policies, and a shift from the formerly predominant ideology of revolutionary nationalism to one of global competitiveness and social liberalism (Cornelius et al. 1994; Aitken et al. 1996).

One of the key sectors in which reforms and modernization were implemented was higher education. Throughout the 1970s, higher education was characterized by sharp increases in student numbers and university staff. Many small and non-professionalized universities transformed into huge, bureaucratically run institutions without much institutional planning or government intervention. Universities operated behind the ‘constitutional shield’ of university autonomy, which restricted direct government intervention in internal university affairs.

The period of ‘unregulated expansion’ was seriously affected by the 1982 debt crisis. While student numbers continued to increase, public funding plunged by one-quarter between 1981 and 1989 (Kent 1993). As a result, universities experienced several acute problems. Some of these problems were a direct consequence of budget cuts, but others—such as academically underqualified or even unqualified staff, factional disputes, union activism and a deteriorating public image—had their roots in longer institutional histories. Since the early 1990s, steps had been taken in Mexico and elsewhere in Latin America to restructure the relationship between the state and higher education. University authorities launched projects of institutional reform and strived to adopt new administrative practices based on concepts such as accountability, evaluation and excellence (Brunner 1994). The BUAP had been a left-wing bulwark since the early 1970s, when university-based political groups clashed violently with an alliance of state, church and private interests and ultimately won control of the university (see Pansters 1998, especially chapters 6 and 7). These conflicts were to result in hostile relations between the BUAP and key actors within Puebla society for many years to come.

From 1970 to 1981 the number of students at the BUAP rose from 8,000 to 25,000. By 1990, the number had increased to 75,000. The ‘open door’ policy and active recruitment of students from middle- and lower-income groups was partly the outcome of the government’s funding strategy based on student numbers. It was also underpinned by the left-wing identity of a *universidad popular, crítica y democrática* (popular, critical and democratic university). The regional political context that pitted the university against outside forces added a further impulse: large student numbers and resources strengthened the university’s bargaining power. A hesitant process of academic professionalization took place. The growing size and complexity of the institution led to the foundation of new administrative bodies, but the professionalization of institutional management failed to keep pace. The process of ‘expansion without institutional reform’ and the resulting administrative disorder was to become an important ingredient in the virtual collapse of the university years later.

At the end of 1989, mounting financial problems reached a climax and forced the university authorities to cut employees’ pay by one-thirds. The university split into two camps, and the situation was only resolved after indirect federal intervention forced the rector to resign. In 1990 new elections were held, the outcome of which propelled the BUAP into a process of profound transformation and transition. A key event was the so-called *Congreso Constituyente* (Constitutional Congress) in the summer of 1991, when the university became the scene of numerous discussions and meetings. The different political groups and factions within the university prepared position papers and proposals and engaged in “foundational debates” about the future of the BUAP during a general assembly or ‘*congreso constituyente.*’^5^ After that, the new university management launched reform projects in almost every domain: the administrative system was reorganized, the teaching branch reordered, the management and payment structure of academic personnel overhauled, and initiatives to promote research launched. Political relations within the institution and between the institutional elites and crucial outside actors changed significantly. A key example of the change within the university was the virtual elimination of the university union as a key political actor within the institution (Pansters 2005). The university’s transition thus reached into the core of academia, altering the positions of university actors and the place of the institution in the wider regional field of forces.

In South Africa, it was the breakdown of apartheid after Nelson Mandela’s release from prison in February 1990 that triggered profound changes (Welsh 2009). In economic terms, after the ANC took up the reins of government in 1994, the interests of South African national and global capital were actively catered to through a macro-economic ‘growth, employment and redistribution’ program, which followed neoliberal recipes. The result is that South Africa, like Mexico, has faced persistent and even deepening inequalities (Bond 2004; Marais 2001). President Thabo Mbeki (1999–2008) tried to combine a neoliberal program with the philosophy of African Renaissance, which re-authenticated African values.

During apartheid, the South African system of higher education was structured along the lines of racial segregation. Segregation had developed from the end of the nineteenth century but reached its ideological pinnacle between 1948 and 1990, when the higher education system attained ‘classical’ apartheid features. This involved the development of three types of universities. The first type encompassed white Afrikaans-speaking universities.^6^ These universities, including that of Stellenbosch, were characterized by a conservative Afrikaner identity and had Afrikaans as their exclusive language. The intricate relationship between Afrikaner professors, administrators, state officials and church leaders was couched in a secret conservative Afrikaner society, the *Broederbond* (Brotherhood), which strived to preserve the unity of the Afrikaner people. The second type was the predominantly white English-speaking university. Although also financially favored by successive apartheid governments, these universities generally took a more liberal position. The third type was the black university, established by the apartheid system for the main black ethnic groups such as Xhosa, Zulu, Sotho, and Venda, as well as for the Indians and so-called Coloreds, a population of mixed descent. These so-called “bush colleges” were mostly located in peripheral areas and were understaffed and underfunded. Confronted with the legacies of racial segregation in higher education, the ANC government wanted higher education to reflect the new political and demographic realities of South Africa. At the same time, universities faced decreasing state funding and increasing student numbers.

The SU was a ‘historically advantaged’ university that sprang from the Stellenbosch theological seminary and the Stellenbosch Gymnasium, institutions created in the middle of the nineteenth century by Afrikaner theologians determined to protect the conservative reformed tradition against modernizing theological trends. Originally established in 1866 as the Gymnasium, it became Victoria College in 1887. In 1913 the Victoria College Council published a memorandum written by three influential Afrikaners, among them Daniel Francois Malan, who became the first prime minister of the apartheid regime in 1948. The 1913 memorandum played an important role in persuading the South African government to establish an independent university in Stellenbosch and in shaping the identity charter of the future university. It reads as follows:

This [i.e. Stellenbosch] is the place from which the *Afrikaner volk* [Afrikaner people] can best realize its ideals and exercise the largest influence in South Africa. It is the best realization the *volk* has yet found of a deeply felt need. It stands for an idea! That’s why it became not merely an educational institution but the symbol and guarantee of its own powerful, growing, and expression-seeking national life. [Thom 1966: 65; translation by the authors]

The memorandum clearly expresses the intimate connections between the institution and Afrikaner identity. In 1916 Victoria College became the University of Stellenbosch. From then on, Stellenbosch played a pivotal role in the development of an Afrikaner intelligentsia closely connected to the politics of Afrikaner nationalism and apartheid. Academic rituals, such as the conferral of honorary doctorates, helped to preserve the Afrikaner identity of the institution. Many prominent apartheid politicians—including four prime ministers (Malan, Verwoerd, Vorster, and Botha)—were closely linked to SU, either as a student, a professor, or as the chancellor. However, the university was also home to more *verligtes* (enlightened) and critical Afrikaner intellectuals (such as philosopher Johan Degenaar), who questioned the apartheid system (see Nash 2009). When Mandela and the ANC assumed power, the University of Stellenbosch faced the most serious crisis in its existence as a *volksuniversiteit* (university of the people—in this case, the Afrikaners). If it was to preserve its presence in the new South Africa, the university was going to have to reformulate the ideological charter of 1913 and to transform its identity.

In sum, at the beginning of the 1990s, both BUAP and the SU faced fundamentally altered political circumstances that pushed for the redefinition of their institutional identities through the elaboration of new institutional narratives and their enactment in distinct public events, ceremonies and rituals. This is what we will be examining in the following three sections.

## Institutional Identity and Lateral Integration

New institutional identities and narratives are developed through public events and rituals which want to constitute and achieve what we call lateral integration, meaning the definition and (symbolic) enactment of new relations of the university with others. We speak of ‘lateral integration’ because we are referring to processes aimed at integrating the university into a changed political environment and establishing alliances with other–previously often antagonistic–societal actors. In what follows we examine these processes for the cases of the BUAP and SU.

### Lateral Integration in Transforming BUAP Identity

As the BUAP faced crucial questions about institutional identity, the university management–elected in 1990 and re-elected in 1993–embarked on the elaboration of a narrative that would explain and legitimize the major transition it had initiated as well as claim a new place and identity for the BUAP. On numerous occasions—in official speeches, press declarations, interviews, university publications, and meetings—Rector José Doger and key members of his administration spoke about what the university had stood for in the past, what it should represent and strive for in the near future, and how this should be accomplished. Gradually, this gave rise to the emergence of a coherent narrative about the history of the BUAP, its project, idea, and its identity.

The core meanings of the grand narrative articulated the shift the university authorities wanted to implement: the institution had to move away from the concept of the *universidad democrática, crítica y popular* and towards the “new university” of *excelencia académica y compromiso social* (academic excellence and social commitment). This was clearly laid out in a strategic development plan called, rather prententiously, the *Proyecto Fénix* (Phoenix Project). A crucial part of this plan involved the construction of a strict dividing line between the “new” and the “old” university (temporal separation). The period before Doger was gradually portrayed as one in which the BUAP was antithetical to academic work and values.

The construction of a line dividing the past and the present was critically underscored by contrasting images of the ways in which the university related to actors in the region. Building a new institutional identity was in part realized by renewing linkages with other institutions and interest groups in Puebla with which the university had long maintained tenuous or even hostile relations. It was a project that was consciously devised and carried out, often in the form of public events. A paradigmatically symbolic occasion in the process of re-articulating the university with regional elites was the public presentation of Doger’s *Proyecto Fénix* on May 13, 1994, which we referred to at the beginning of this article. Invited to the presentation were the highest government officials–such as Governor Manuel Bartlett, Mayor Rafael Cañedo, and the leader of congress–presidents of business associations, as well as a representative of the archbishop. In a 40-minute slide show, Doger spoke about the major components of the development plan. The presentation was, however, entirely framed by the broader political objective of obtaining the recognition from BUAP’s one-time foes. This was plainly clear from the title of the meeting: “Development Plan of the BUAP. A new relationship with society.”

After Doger outlined the development plan, he invited business leaders to work together with the BUAP, stating: “we don’t deny the confrontations between the university and other sectors in society in previous years … but now these problems are over and that is why a commitment should prevail, a co-responsibility between university and society” (Universidad 1994: 7). Finally, Doger announced the establishment of several councils and advisory organs that would function as channels for the new relationships between the BUAP and society, one of which would be chaired by a prominent member of Puebla’s Mexican-Lebanese business community (Doger himself is of Mexican-Lebanese descent). At the end of the meeting, Governor Bartlett praised the “political will and courage” of Doger’s team and promised full support, “while respecting university autonomy” (Universidad 1994: 7). However, within the university itself, other narratives emerged that contested the new approach of the university elite. One important alternative narrative was based on the resentment of the university’s growing ties with regional elites, and questioned the need to impose a sharp break from the past. Rather than being seen as proof of a promising new beginning, much-publicized public events—such as the launch of the *Proyecto Fénix*—were perceived as spectacles in which university autonomy was being sold out to the regional bourgeoisie and to the state.^7^

Doger continued to reiterate his project of lateral integration through mandatory annual addresses in which he publicly presented to the University Council a review of his policies and the university’s current situation. A recurring administrative event with its standardized and repetitive symbolic features, the annual address (*Informe Anual*) underwent a profound transformation after Doger became rector in 1990. While its basic formal features remained largely unchanged, the form they acquired and the context in which they were acted out changed considerably. With its impressive colonial baroque halls, the University of Puebla gradually turned into a podium for a non-university audience. The yearly addresses thus made visible the broader transformation whereby the university authorities became tied up with the state, the church, and national and regional political and economic elites. It was clear from the onset that Doger used his annual address as a platform to act out his policies symbolically.

The performance of Doger’s first annual address succeeded in scoring a major symbolic upturn with the attendance of the Archbishop of Puebla, Rosendo Huesca Pacheco. It was the first time in more than twenty years that a high-ranking cleric had visited the university. Huesca Pacheco himself knew that his presence was controversial and declared that it should not be interpreted as mingling in university affairs. He thereby touched on a core value of Mexican public higher education, that of university autonomy. The Doger clan’s political and ideological project of lateral integration and its insistence on a temporal break with the past were bound to recast the meaning of university autonomy. From 1991 onwards the archbishop was to consistently attend the rector’s annual address.

### Lateral Integration in Transforming SU Identity

In post-apartheid South Africa, the transition in higher education was closely related to debates about identity, including how the Afrikaners should cope with the loss of political and identity power after 1990. Different narratives of Afrikaner identity were to develop over time (Steyn 2001). At one end of the spectrum, there is the narrative about whites as the victims of recent developments, with at the other end the narrative about whites willing to accept the ambivalence of whiteness and African belonging. The language of Afrikaans has played a prominent role in these narratives. It is no surprise, then, that discussions about repositioning the institutional identity of the formerly Afrikaner University of Stellenbosch have centered on the issue of language.

After the end of apartheid, the University of Stellenbosch came under external pressure to reposition itself in the new South Africa and to cope with the loss of its former prominence and intimate links to the apartheid regime. In this rapidly changing environment, it became clear that the 1913 memorandum had become outdated, and that it was necessary for the university to redefine its “idea” and raison d’être.

It took the university more than ten years to formulate a new strategic document in which it presented itself as a university in a new regional, national, and global setting. In a council meeting in March 2000, the university council formally approved the *Strategiese Raamwerk vir die Eeuwisseling en Daarná* (Strategic Framework for the Turn of the Century and Beyond). This document defined the university at three interrelated levels. The first concerned the level of the global playing field of universities dominated by a neoliberal narrative of excellence, accountability, and marketization. The second acknowledged the new post-apartheid South African political and cultural playing field, with key themes such as “diversity” and “access.” The third level hinted at the predicament of an increasingly fragmented Afrikaner community that lay at the foundations of the university. The *Strategiese Raamwerk* stated that “the university acknowledges its historical ties with the people from whom and communities from which it arose … therefore the University commits itself to be language-friendly, with Afrikaans as the point of departure.” In other words, identity was closely associated with language. In repositioning the previously Afrikaner university in post-apartheid South Africa, the *Strategiese Raamwerk* is a foundational document that gradually became the new charter of the University of Stellenbosch. At the same time the ‘genealogy’ of the white Afrikaner narrative, including the position of Afrikaans as an identity marker, had to be addressed.

Shortly after the document was formalized and adopted, in 2002, Chris Brink became the new rector of Stellenbosch. Not part of the tightly knit Matie community, Brink’s appointment came at the defeat of a candidate from Stellenbosch’s inner circle. As part of the selection process, Brink formulated his personal vision for the university. He cautiously touched upon the particularities of the ‘Afrikaner’ university, typifying Stellenbosch as “the natural home and mouthpiece of a minority group that realizes that its survival depends on reaching out, not on exclusivity” (Botha 2007: 44). Brink utilized the notion of *diversity*, a keyword in the ideology of the rainbow nation, in which people from different racial and social backgrounds live together in harmony. There was an urgent need to diversify students and staff at the university. In 2002, 85 percent of undergraduate enrolments (12,698 in total) were white, whereas black students made up only 2.6 percent. The largest group of non-white students was colored (12 percent). Although diversity was supposed to open up the university to other groups, between 2002 and 2006, black undergraduate students came to form only 4.3 percent and colored students 15 percent of the total student population (Botha 2007: 33). Clearly, the genealogy of the University of Stellenbosch remained powerful enough to thwart the establishment of a far-reaching diversity framework.

In response, a different understanding of diversity increasingly came to the fore in the debate on institutional identity. In a study of Stellenbosch, anthropologist Cees van der Waal (2002: 24–25) distinguished between “an inclusive and critical approach (affirmative diversity), and an exclusive and essentialist approach (difference diversity).” Affirmative or inclusive diversity linked seamlessly with the notion of the *rainbow nation*, and had also inspired the official university documents and Brink’s personal vision. But passionate discussions at Stellenbosch increasingly focused on language as the symbolic marker of identity, thereby introducing the concept of difference diversity and its connotations of separatism and essentialism. Policies of inclusive diversity encountered growing opposition from parts of the Afrikaner community, who claimed the alleged constitutional right to a minority position, including within its own university, in which Afrikaans would remain the essential identity marker. The central question became whether Afrikaans was to remain the primary institutional language. In December 2002, the university council adopted the *Taalbeleid van die Universiteit Stellenbosch* (Language Policy of the University of Stellenbosch) which expressed a commitment to the use and sustained development of Afrikaans as an academic language, albeit in a multilingual context.

Even after the formal endorsement of the language policy, discussions continued as Afrikaans became the symbol for Afrikaner academics and Stellenbosch alumni who closely identified with what Steyn and Foster called “resistant white discourses” (Steyn and Foster 2008: 26). Afrikaans was seen as *the* symbol of the Afrikaner community’s predicament. However, resistant white discourses redefined the meaning of ‘Afrikaner community’ by referring no longer to the *white* Afrikaner community exclusively but to a broader community of Afrikaans-*speaking* people. This was a clear attempt to incorporate the Afrikaans-speaking colored population in the Western Cape.

Only one year after having started his second term, Chris Brink resigned unexpectedly in 2007 and left for the UK. The new rector, Professor Russel Botman (1953-2014), was a prominent theologian in South Africa and a member of the colored community. In his inaugural address, Botman replaced his predecessor’s contested project of critical diversity with a vague but nonetheless effective appeal to a “pedagogy of hope,” thereby paving the way for a process of de-escalation. As a result, the language debate began to lose its ideological features.

## Identity and the Politics of Honorary Degrees at the BUAP and SU

A prominent scholarly ritual in most universities is the granting of honorary degrees, which confirms (or reconfirms) the distinctiveness of the academic community, and symbolically regulates the incorporation of individuals into the local academic community (Manning 2000). With clear features of a rite of passage, and with the considerations in our conceptual framework in mind, we employ the concept of ritual here, albeit always with the purpose of relating it to processes of articulating and enacting new institutional narratives and identities. At the same time, it can also enhance the alignment (or re-alignment) of the university with other sectors in society as the recipients of honorary doctorates often come from the (external) worlds of the arts, politics, and industry. The rituals of granting honorary degrees can therefore enact both continuity and transformation of the university.

### Granting Honorary Degrees at the BUAP

Although either the rector or the University Council at the BUAP can take the initiative to grant an honorary degree, in practice the rector proposes candidates to the University Council. For many years, the granting of honorary degrees was uncommon practice at the BUAP. During the period of left-wing dominance of the institution between 1976 and 1988, only 11 degrees were granted (compared with 34 between 1990 and 2002). In the spirit of the *universidad crítica, popular and democrática*, several degrees were driven by political considerations. Such was the case of the honorary degrees granted to the Chilean communist leader, Luis Corvalán (1976); the Nicaraguan Sandinista commander, Tomás Borge (1981); the left-wing Uruguayan general, Liber Seregni (1983); the wife of former Chilean president Salvador Allende (1988); and, finally, to the grand old man himself of the university left and former rector of the BUAP, Luis Rivera Terrazas (1984).^8^

During these years, the ritual granting of degrees was a small-scale event mainly directed at the university community. Things were to change rapidly, however, when José Doger became rector.^9^ In his first (three-year) term, seven honorary degrees were conferred; and in his second (four-year) term, thirteen degrees were awarded. Most went to distinguished scholars such as Mexican sociologist González Casanova, albeit with some notable exceptions. The first honorary degree awarded by Rector Doger went to Hector Azar Barbar, a nationally known and prominent theatre maker with close relations to the regional and national political elites and originally from Puebla. Azar would later become the Secretary of Culture of the State of Puebla under Governor Bartlett. The selection of Azar was the beginning of a trend that deepened during the rest of the 1990s: the academic ritual of granting *doctorados honoris causa* became part of the strategy of what we have called lateral integration. The selection of candidates from the worlds of academia, politics, and literature was guided increasingly by the possibilities of building new relations with key actors in society (for an interesting comparative study about the politics of honorary degrees at the universities of Oxford and Cambridge, see Hefferman and Jöns 2007). Scholarly grounds for granting such degrees seemed to become less important.

The trend to convert the ritual of granting honorary degrees into an event that would project the BUAP—and the rector himself—onto the national scene drew significant attention from the media. Important public personalities were invited to attend these events, which furthered (national) media attention and put the BUAP and its leaders in the spotlight. A clear symbolic expression of the deepening relations between state and university elites was when, in 1994, the daughter of Vicente Lombardo Toledano, a key architect of Mexico’s one party regime, who had passed away in 1968, received an honorary doctoral degree on his behalf from the governor of Puebla and not from the rector.^10^ In other words, the state governor momentarily assumed the academic authority of the rector. The appeal to regional and national political and cultural elites became even more accentuated after Doger left office and his nephew, Enrique Doger, took over. It is particularly interesting to note that several honorary degrees were granted to internationally known figures from the literary arts: poet Germán List (postmortem, 1998); essayist Carlos Monsiváis (2000); and writers Carlos Fuentes (2001), Elena Poniatowska (2002), and Angeles Mastretta (2003). Another renowned figure honored with a doctoral degree was Spanish judge Baltasar Garzón, who obtained international prominence with his much-publicized legal case against Augusto Pinochet in 1998. Lavishly funded, these events became occasions to be seen with global celebrities such as Fuentes and Garzón and to build networks between *universitarios* and people from the worlds of politics, business, and the media. Apart from attracting broad media attention, this further strengthened the trend to move away from the domain of academic scholarship and confirmed that the university was increasingly becoming a platform for the extra-university interests of its leaders than its prime area of concern.^11^

There is also a more explicit political dimension to the BUAP’s ritual of granting honoris causa degrees, one related to the narrative of the division between the ‘old’ left-wing and politicized university and the new ‘non-political’ university of academic excellence. A key moment in the symbolic articulation of the official discourse of depoliticization was precisely the granting of two honorary doctorates in 1995. During a meeting of the University Council, Rector Doger recommended to its 146 members the conferment of three honorary degrees—the first one to Ruíz Reyes, a nationally and an internationally renowned specialist in hematology from Puebla, was unanimously accepted by the council, except for one abstention (for more information about Ruíz Reyes, see BUAP 1995a). The two other proposals were far more contested: one concerned an honorary doctorate to Fidel Castro and the other to Puebla’s Archbishop Rosendo Huesca Pacheco. That these proposals were submitted by the rector to the council *simultaneously* contains much symbolic and political meaning. What appeared to be a “package deal” for the University Council was an example of symbolic engineering designed to articulate the changing ideological context of the university and to court distinct audiences. The choice of Ruíz Reyes payed lip service to the official discourse of ‘academic excellence’ and appeased the university’s scholarly community. The case of Huesca Pacheco underscored the rector’s strategy to reconstruct relations with one of the university’s one-time foes in the region, while a degree for Castro would placate many members of the BUAP community, including Doger himself, who had long looked upon the Cuban commander as one of Latin America’s most important protagonists and icons. The council widely approved Castro’s honorary degree, with one vote against and ten abstentions. However, the case of Huesca proved more controversial with 22 votes against and 36 abstentions, that is, almost 40 percent of the council members expressed serious reservations (*Acta de la VIII reunión del H. Consejo Universitario*, September 26, 1995, Archivo Secretaría General BUAP).

To understand the deeper symbolic meaning of this event, we must recall that the origins of the BUAP left-wing camp lay in a student movement in support of Castro’s revolution and against the U.S. attempt to oust him from power in April 1961. From that moment onwards, Cuba and Castro became potent symbols at the university. At the same time, the Catholic Church in Puebla represented the most vociferous opponent of communism, with its opposition directed especially at Puebla’s public university. For years, Huesca’s predecessor, Márquez y Toriz, was perceived by many in the BUAP as one of the university’s fiercest adversaries. During the 1960s and 1970s, Castro and Márquez y Toriz thus symbolized the antipodes of a profound political, cultural and moral antagonism. Doger’s decision to propose bestowing honorary degrees on both men simultaneously can only be comprehended in the light of this particular institutional history. Against this background, it also becomes understandable that in his speech during the ceremony in which Huesca received his degree, Doger spoke of the virtues of pluralism. Pluralism and the idea of the perfectibility of man, according to Doger, fall into the fold of ‘post-ideological’ politics (see BUAP 1995b: 29–32). Dressed in civilian clothing, the archbishop himself endorsed the idea of lateral integration, suggesting that the university abandon its position of (scholarly) isolation and embrace the institutions that surround it (BUAP 1995b: 36; see also Ortiz Ortiz 1995: 14–15).

### Granting Honorary Degrees at the SU

In contrast to the BUAP, the University of Stellenbosch had always pursued an active policy of granting honorary doctoral degrees. It employed this traditional academic ritual to strategically strengthen its political, economic, academic, and cultural ties to the worlds of politics, business, and the arts. During apartheid, two of its major architects (Malan and Verwoerd) received honorary degrees. Malan was a former student and chancellor of the university, and Verwoerd began his career as a professor in social work and industrial psychology there. The last prime minister of the apartheid era, F. W. De Klerk, received an honorary degree in December 1990, the year Mandela was released from prison.

An analysis of the honorary degrees after 1995 immediately reveals an attempt to create linkages with different segments of society. Already in 1996, Mandela, the political and moral leader of the new South Africa, was awarded an honorary degree. This was followed in 2000 by an honorary degree for Walter Kamba, the black legal scholar, former vice-chancellor of the University of Zimbabwe, and member of the first elections commission in South Africa. Trevor Manuel, the successful ANC Minister of Finance, received a degree in 2001, together with two previous finance ministers of the former regime (Derek Keys and Chris Lieberberg). As an internationally renowned champion of the anti-apartheid struggle, Bishop Desmond Tutu was honored in 2002. Mamphela Ramphele, the first black female vice-chancellor of the University of Cape Town and intimately engaged with Steve Biko, received an honorary degree in 2003. The University of Stellenbosch also honored critical academics such as theologian Jaap Durand (2002); Jakes Gerwel, former vice-chancellor of the leading anti-apartheid and former ‘colored’ University of the Western Cape (and then head of Mandela’s cabinet); and the writer and poet Antjie Krog (2004). Thus the (symbolic) repositioning of the university in a changing national environment was ritually enacted and brought about through the awarding of honorary degrees.

At SU, the graduation ceremony takes place in the D. F. Malan Hall, a huge square building situated on the sports ground of the university. The ceremony of conferring honorary doctoral degrees commences with the solemn entrance of the academic procession into the hall. The chancellor welcomes the attendees and formally confirms the congregation, thereby instantiating the notion of the academic community, which is followed by the singing of the national anthem (parts of which, after 1994, are now sung in Xhosa, Zulu, Afrikaans, and English). After a prayer the degrees are formally conferred. In this way, values connected with academia, nation and religion are interconnected and performed symbolically, clearly giving the ritual a magico-religious dimension.

In 2004 South Africa’s president and ANC leader, Thabo Mbeki, was conferred an honorary doctorate in commercial sciences. Mbeki stressed Stellenbosch’s “great educational and symbolic value for Afrikaans speakers.” Mbeki concluded his speech symbolically by addressing the crowd in Afrikaans with the words quoted at the beginning of this article: “And there I will proudly say to the world, that I too have become, honoris causa, a scholar of Matieland.” The politically sensitive issue of Afrikaans, the language of the former oppressor, was contextualized skillfully by linking it to the “language of inclusive diversity” while the university succeeded in symbolically incorporating Mbeki into the community of this previously Afrikaner university.

Conferring an honorary doctorate on Mbeki could easily be interpreted as a deliberate move by the university to liaise with representatives of the highest circles of power in post-apartheid South Africa. The awarding of an honorary doctorate to Bram Fischer in 2004, however, proved to be far more delicate for the Afrikaner community. Fischer was a distinguished lawyer of Afrikaner descent who had led the defense of Nelson Mandela during the Rivonia Trial (1963–64). He had joined and for some time headed the South African Communist Party. In 1964 he was arrested and charged with treason and sentenced to life imprisonment. Fischer contracted cancer while in prison and was released on humanitarian grounds in April 1975, shortly before his death.

The proposal to honor Bram Fischer postmortem provoked an intense debate. Some opponents pointed to Fischer’s Marxist (and allegedly Stalinist) sympathies. More importantly, the Fischer debate became closely intertwined with the identity debate at the university. Supporters felt that it was right to confer an honorary degree on a member of the Afrikaans community who had stood up against apartheid. Opponents argued that Fischer had unambiguously turned away from his Afrikaner community to fight the Afrikaner-based regime—if necessary with force. As a communist, he had confronted the apartheid regime which had warned against the threat of the ‘*Rooi Gevaar*’ [Red Danger] taking over Africa. As one outspoken participant in the debate put it, the debate was a ‘battle of symbols’ (Annie Gagiano n.d.). Although opponents, among them a group of former academics still clinging to a neo-Afrikaner identity for Stellenbosch, attempted to derail the initiative, an eventual compromise accepted the honorary degree while acknowledging the existence of dissent. In a solemn event in December 2004, the honorary doctorate was conferred in the presence of Fischer’s daughter.

## Transition as Comprehensive Ritual

Our analysis of the launch of the *Proyecto Fénix*, and the yearly *Informes* at the BUAP, and the publication and ritualized use of the *Strategiese Raamwerk* at SU as well as the granting of honorary degrees at both universities, has revealed different aspects of the political and cultural transitions at these universities. What can we learn about these political and symbolic transitions in the BUAP and the SU if we examine these public events, ceremonies and rituals from a comprehensive perspective able to identify meaningful connections between them? In other words, can the perspective of transition as a (comprehensive) ritual process, such as proposed by Edles (1998)—comprising the elements of separation, liminality, and reaggregation—contribute to a better understanding of the meanings of specific public events and their interconnections?

At the BUAP the moment of separation would have to be the spectacular collapse of the university power structure at the end of 1989. This was later portrayed as a key moment of separation in BUAP’s history and social position. Doger’s narrative about the period before his reform project as one of “obscurantism” is constitutive of the symbolization of separation and “a new beginning.” In fact, the construction of a new organizational identity along the lines of a diachronic otherness was built largely around this sharply delineated moment of rupture. The correspondence between historical and social separation is corroborated by the simultaneity of the construction of a diachronic otherness and lateral (social and political) integration, allowing the BUAP to acquire a new position in Puebla’s social and political system.

The event that most compellingly symbolized the phase of liminality in the transition of the BUAP is that of the previously mentioned *Congreso Constituyente* in 1991, when University Council members gathered at the neutral terrain of a hotel to discuss more than one hundred proposals.^12^ Although Rector Doger and his advisors played an important role during the deliberations, the meeting was widely perceived as an occasion in which everyone had an opportunity to put forward ideas. From the outset, Doger was very clear about his political objective in stating that “at the BUAP, those historic vices that were the cause of the development of the clientelist, populist and pseudo-democratic processes of universal, direct and secret voting, should not be repeated” [translation by authors].^13^ The *congreso* had the communal and homogenizing effects so characteristic of the state of liminality.

This foundational event produced a legal and symbolic framework within which a more “substantial” project was developed that condensed the end state of reaggregation, best symbolized by the launching of the *Proyecto Fénix* in 1994. There can hardly be a better metaphor for a new beginning than the “Phoenix from the flame.” With its already matured narrative of temporal separation, the launch of the *Proyecto Fénix*—an elaborate reform project for the institution and a symbolization of lateral political integration—as well as the public events of the *Informe Anual* and the granting of honorary degrees obtain their full meaning only if they are understood as key moments in a process in which the new social and symbolic state of the university was consummated, that is, as a last phase of a prolonged ritual process of institutional transition. A comprehensive perspective on these separate and specific public events reveals their constitutive role in bringing about a profound political and symbolic transition of the university and in generating a new institutional identity.

However, an emphasis on transition and change may mask the complexity of these university arenas. In the case of Stellenbosch, the process of transition was forced upon the university by the dramatic changes that South Africa was experiencing with the end of apartheid. Until then, the university had been intimately connected to the apartheid regime, partly embodied by a strategic policy to confer honorary degrees to key persons in Afrikaner politics and business. The honorary degree granted to the last Afrikaner prime minister in December 1990 perhaps symbolizes the early beginnings of separation. The “idea” of the University of Stellenbosch dating from 1913 could no longer serve as its “charter of identity.” Hence, the phase of institutional liminality was characterized by the university’s laborious efforts to cope with the loss of power (both direct and indirect) and to reposition itself in a post-apartheid state and society. The reorientation of the university involved passionate debates about its position on different playing fields. One of them, the Afrikaner community, became a battlefield of competing institutional identities and focused increasingly on language. Afrikaans became *the* symbolic marker of the opponents of a radical transformation of the university in the new South Africa. For Stellenbosch, liminality was characterized not so much by the homogeneity and communality we observed with BUAP’s *congreso constituyente* but more by fragmentation and contestation. The institutional narrative of inclusive diversity—propagated by the university management under Rector Brink (2002–07) and mostly seconded by the university’s senate and council—was aimed to rebuild a university in a new regional and national context. However, it was opposed by a counter-narrative that reaffirmed and re-authenticated the identity of an Afrikaans-speaking community. By deliberately including Afrikaans-speaking coloreds, this counter-narrative focused on the most important symbolic marker of their community, namely, the Afrikaans language. A process of reaggregation was only initiated when Brink’s successor (Botman) constructed a narrative around a pedagogy of hope, harmony, community, and continuity that ushered in a period of relative rest.

## Conclusion

We began this article with a reflection on the relationship between public events, ceremonies, academic rituals, and institutional identity and transition. We pointed at Kertzer’s argument that ritual can play a key role in political conflicts, revolutions, rebellions, and, more generally, processes of change and transition. However, following Handelman, we intentionally broadened our conceptual scope with the notion of public events, which enables us to examine events that did not necessarily operate according to the logic of ritual processes, but that did play a role in effectuating institutional transition and identity formation. We also set out investigating our cases with the help of the framework of transition as comprehensive ritual. Our starting point was that during the 1990s and the early 2000s the BUAP and SU both underwent such processes as part of larger societal transitions powered by neoliberal reform and democratization. Several conclusions can be drawn from our analysis.

First, the very idea of transition presupposes a point of rupture. Several university public events and rituals played a crucial role in bringing about and representing this rupture. Important institutional ceremonies and events, such as the *Informe Anual* and the launching of the *Proyecto Fénix* at the BUAP, provided ample symbolic opportunities and techniques to do so, for example, by inviting guests such as the state governor, who only shortly before would not have been welcome at the university, and by giving them high-profile treatment, such as the granting of an honorary doctorate to the Archbishop of Puebla. These public events themselves not so much reflect but constitute points of rupture and identity formation. The publication of the *Strategiese Raamwerk* as the new foundational charter of SU triggered efforts to redefine the raison d’être of the university, as it found itself in a new (inter)national playing field. More so than at the BUAP, the genealogy of the institution’s historical narrative continued to be powerful. Symbolic tokens of transition—such as the official university policy to install signposts on campus in Xhosa, English and Afrikaans as symbolic markers of a new institutional identity—could not mask the university’s failure to implement its policy of inclusive diversity. It is our contention that the noticeable difference between our two case studies in this respect is explained by the fact that whereas at the BUAP the primary source of rupture came from within (in the form of the university’s political and financial collapse), albeit conditioned by forces from without, at SU the main source of rupture came from without (with the collapse of apartheid).

Second, notwithstanding these differences, we found that in both cases a new politics of identity produced its own counter-politics and identifications. In Puebla, some groups at the university protested against the construction of a radical dividing line in the institution’s own history and the conscious repudiation of the university’s former left-wing activism and role in society. In Stellenbosch, the articulation of a politics of identity closely tied to Afrikaans, thereby expanding the Afrikaner community to a broader Afrikaans-speaking community, enabled the institution to symbolically perform lateral integration, while at the same time reining in a more profound transformation of the university. Recent events on campus, with black students demanding fundamental transformation, illustrate the failure of this policy.^14^ A comparative study of public events and ritual processes makes clear that complex local histories and constituencies consistently and decisively mediate and transfigure the identity projects initiated from above in response to inside or outside sources of change and pressures for lateral integration.

Third, our analysis has hinted at the growing importance of the figure of the rector himself. At the BUAP, the rector’s centrality in numerous and glamorous public performances and rituals constitutes and represents a process of political centralization. Renewed political and symbolic centralization—to a degree unknown before—also meant the rebirth of effective and strong leadership. In a way, the entire transition can be construed as the reconstitution of personalized executive leadership to the detriment of institutional counterweights. The transition has certainly not produced an unambiguously democratic governance system and political culture at the BUAP. At the University of Stellenbosch, the new foundational charter of the *Strategiese Raamwerk* came to serve as a guide for institutional transformation. It became the new *idea* of the university. Not “born and raised” in the Stellenbosch Matie community, Rector Brink unmistakably put his personal “transformational” stamp on the debate about the implementation of the *Strategiese Raamwerk*. He indisputably was the central—and controversial—figure in the institution. However, his rectorship was marred by tensions between the imperatives to reposition the university and the institutional genealogy of Stellenbosch. His successor invoked the vague but tempting idea of “a multicultural university with a pedagogy of hope for Africa” (Botman 2007).^15^ A new institutional discourse of harmony and hence reaggregation emerged at the University of Stellenbosch.

Fourth, we demonstrated the role of public events, ceremonies, and rituals in the politics of institutional identity. In Puebla, they enacted and performed a shift from a synchronically drawn boundary between university and external actors toward a diachronically drawn boundary between institutional identity and otherness in the history of the institution itself. Symbolically, this meant that the narrative of the *universidad popular, democrática y crítica* was replaced by one that centered on academic excellence and tied into neoliberal discourses of accountability and efficiency. Politically, this made possible the (re)construction of relationships between the university, government, political elites, business and the church in Puebla and Mexico City. That former rectors José and Enrique Doger became prominent members of the government elite in Puebla soon after leaving office epitomizes this political effect. It also refutes the claim that the transition achieved a measure of depoliticization. The outcome of the transition was certainly a recasting of the political—in terms of new political relations, hierarchies and symbols—but not an ousting of the political. That claim is itself ideological and thus deeply political. Moreover, the basic logic and culture of personalistic political practices remained entirely intact and were even strengthened (see also Pansters 2005). At the University of Stellenbosch, the academic ritual of conferring honorary doctorates has remained unchanged; but as in Puebla, the reservoir of actual and potential recipients of honorary doctorates has changed. New external political networks were constructed in part through the deliberate use of academic rituals which, paradoxically, contributed more to the continuity with the apartheid era than the transformation of the university’s identity in post-apartheid South Africa. The *Strategiese Raamwerk* embodies new forms of lateral integration without fundamentally transforming the institution.

Therefore, and finally, putting too much emphasis on public events and rituals in terms of transition and change unduly neglects their ability to construct and reinforce continuity and stability.

## Footnotes

1 “Matieland” is the designation of the academic community of the University of Stellenbosch.

2 See https://www.youtube.com/watch?v=nPV3klYJ25Q, accessed 16 October 2015.

3 Anthropological theorizing about ritual has been particularly relevant in this regard. See e.g. Baringhorst (2001), Bell (1992), Kertzer (1988, 1996).

4 We thank an anonymous reviewer for sharpening our views on this point.

5 The use of this name conjures up many meanings deeply rooted in Mexican history, such as the *congreso constituyente* during the revolution that led to Mexico’s progressive 1917 Constitution.

6 People claiming to be *Afrikaners* were mobilized around the idea of being an ethnic group in South Africa that descended from Dutch, French, and German settlers. Their native tongue is Afrikaans. Afrikaans derives primarily from seventeenth-century Dutch, as well as from other languages. South Africans of British descent can be seen as a separate ethnic group from Afrikaners. Their first language is English.

7 This claim is based on a large number of interviews throughout the university and on an extensive study of local newspapers and university publications.

8 Information about these honorary doctorates was retrieved from the press as well as original documents from the university archives.

9 During the politically unstable years between 1988 and 1990, no honorary degrees were awarded.

10 *El Sol de Puebla*, 20 October 1994.

11 Between 1991 and 1997, José Doger granted twenty honorary degrees, four of which were to non-academics; between 1997 and 2003, Enrique Doger granted seventeen honorary degrees, seven of which were to non-academics.

12 *Congreso Constituyente* refers to meetings and deliberations oriented towards drawing up a new constitution or a ‘foundational document’ of a particular institution or organization.

13 Doger made this statement to the region’s most important newspaper *El Sol de Puebla*, 11 September 1991.

14 See e.g. the opinion of the Open Stellenbosch Collective, which is a movement of predominantly black students and staff at the University. http://www.dailymaverick.co.za/article/2015-04-28-op-ed-open-stellenbosch-tackling-language-and-exclusion-at-stellenbosch-university/. Accessed 16 October 2015.

15 When finishing this article the authors were informed that Professor Russel Botman had suddenly died on June 28, 2014. On July 2nd, only a few days after his sudden death, an article was published in the *Daily Maverick* entitled ‘Revealed: Professor Botman's torrid final week’ about the tensions at campus with regard to his project of institutional transformation: “The point is that Botman was attempting to tread a delicate path between the views of an apparent minority of influential council members and the need for transformation at the almost 150-year-old university with all its history and institutional memory.”
